# Relationship between serum HIF-1α and VEGF levels and prognosis in patients with acute cerebral infarction combined with cerebral-cardiac syndrome

**DOI:** 10.1515/tnsci-2022-0295

**Published:** 2023-08-18

**Authors:** Qing Chang, Hongna Liu, Ermiao Zhang, Qian Xue, Aixia Song

**Affiliations:** Department of Neurology, First Affiliated Hospital of Hebei North University, Zhangjiakou, 075000, Hebei, China; Department of Cardiovascular Medicine, First Affiliated Hospital of Hebei North University, Zhangjiakou, 075000, Hebei, China

**Keywords:** acute cerebral infarction, cerebral-cardiac syndrome, HIF-1α, VEGF, prognosis, relationship

## Abstract

**Objective:**

This research was conducted to discuss the recent prognosis of patients with acute cerebral infarction (ACI) combined with cerebral-cardiac syndrome (CCS).

**Method:**

Eighty-seven patients with ACI were selected, which were divided into the ACI group (52 patients) and the CCS group (35 patients) according to whether the CCS was combined, and another 30 health controls were selected as the control group. Serum hypoxia-inducible factor (HIF)-1α and vascular endothelial growth factor (VEGF) levels of subjects in each group at the 1st day, the 3rd day, and the 7th day after admission were measured by enzyme-linked immunosorbent assay. After discharge for 30 days, the National Institutes of Health Stroke Scale (NIHSS) and the modified Rankin Scale (mRS) score were utilized to evaluate the prognosis of patients. The role of serum HIF-1α and VEGF levels in the prognosis of ACI combined with CCS patients was assessed by receiver operating characteristic curve and the binary logistic regression analysis.

**Results:**

Higher serum HIF-1α and VEGF levels were observed in the CCS and ACI groups versus the control group, and the levels of which were even higher in the CCS group in comparison to the ACI group. According to the prognosis of the NIHSS score, fasting blood glucose (FBG), Acute Physiology and Chronic Health Evaluation II score, creatine kinase-MB (CK-MB), and HIF-1α and VEGF levels at the 7th day of admission were higher while Glasgow coma scale (GCS) score was lower in the poor prognosis group than those in the good prognosis group, and the area under the curve (AUC) of serum HIF-1α and VEGF levels was 0.895 (95% confident interval [CI], 0.786–1.000), and 0.855 (95% CI, 0.731–0.980). According to the prognosis of the mRS score, FBG, CK-MB, and HIF-1α and VEGF levels at the 7th day of admission were higher while GCS score was lower in the poor prognosis group than those in the good prognosis group, and the AUC of serum HIF-1α and VEGF levels was 0.850 (95% CI, 0.722–0.979) and 0.901 (95% CI, 0.798–1.000). The results of the binary logistic regression analysis revealed that HIF-1α and VEGF levels may be independent risk factors influencing the prognosis of ACI combined with CCS.

**Conclusion:**

Serum HIF-1α and VEGF have a good predictive value for assessing the recent prognosis of patients with ACI combined with CCS, which could be independent risk factors influencing the prognosis of disease.

## 1 Introduction

Acute cerebral infarction (ACI) is known as a cerebrovascular disease frequently found in middle-age and elderly people in clinical practice, and this disease has a high incidence rate, disability rate, and mortality rate [1]. Cardioembolic stroke is the subtype of ischemic infarct with the highest in-hospital mortality. The short-term prognosis of patients with cardioembolic stroke is poor compared with other ischemic stroke subtypes [2]. Early prediction of long-term adverse reactions of ACI patients based upon prognostic factors helps to guide early intervention to improve the quality of life for ACI patients [3]. Cerebral-cardiac syndrome (CCS) resulted from the interaction between the heart and the brain is a type of systemic damage causing by the central nervous system diseases [4]. CCS can be caused by the direct induction of certain areas of the brain, leading to sympathetic or parasympathetic responses or neuroendocrine responses [5,6]. The development of CCS subsequent to ACI contributes to prolonged hospital stay, significant elevation in hospitalization costs, worsened functional outcome, and even death [7]. With decades of advances in medical science and technology, there is increasing evidence further recognizing the importance of the brain–heart connection, now referred to as neurocardiology [8] and cardiac dysfunction secondary to brain injury is now referred to as CCS [8,9]. In addition, CCS significantly affects the prognosis, morbidity, and mortality of stroke and is the second leading cause of death after cerebrovascular disease [8]. Therefore, early identification and prevention of CCS are very important in ACI patients.

Recently, emerging evidence has focused on the functions of vascular endothelial growth factor (VEGF), hypoxia-inducible factor (HIF), brain-derived neurotrophic factor, and nerve growth factor signaling pathways in neuroprotection [10]. HIF-1α is a vital transcription regulator for diverse angiogenic factors (VEGF and Netrin-1) under hypoxia, which could control the biological effects to adapt to hypoxia for systems, tissues, and cells [11]. Importantly, upregulated HIF‐1α is observed in various types of human tumors and their metastases and is associated with a more aggressive tumor phenotype [12]. Meanwhile, HIF‐1α has also been regarded to be an important factor modulating apoptosis after brain injuries, such as cerebral ischemia and traumatic brain injury [13]. VEGF is a neurotrophic factor that has comprehensive effects on neural and endothelial cells, and it could increase brain capillary density, reduce neuronal death, and enhance post-ischemic blood–brain barrier integrity under pathological conditions [14,15]. Although HIF-1α and VEGF have been widely known to be elevated in brain diseases, but the exact roles of HIF-1α and VEGF in ACI combined with CCS are still being unsolved. In this study, we examined the roles of HIF-1 and VEGF levels in determining the prognosis of ACI combined with CCS patients.

## 2 Materials and methods

### 2.1 Participants

Eighty-seven ACI patients who had 48 h of hospitalization and received treatment in the Department of Neurology of First Affiliated Hospital of Hebei North University from June 2019 to June 2021 were retrospectively analyzed. These patients were divided into the ACI group (52 patients) and the CCS group (35 patients) according to whether CCS was combined. Inclusion criteria were as follows: all patients with cerebral infarction met the diagnostic criteria of acute ischemic stroke [16] and confirmed by head computed tomography or magnetic resonance imaging. The diagnostic criteria of CCS were as follows: no history of coronary heart disease before the onset, secondary cardiac damage manifestations during the onset of ACI, including ECG changes, acute heart failure or even pulmonary edema, myocardial ischemia or even myocardial infarction, and abnormal myocardial enzyme spectrum [7,17]. Exclusion criteria were as follows: patients with a history of coronary heart disease, myocardial infarction, arrhythmia, and heart failure before the onset of the disease; patients with severe underlying diseases such as liver and kidney insufficiency and lung disease; patients with a history of malignant tumors; and patients with severe infectious diseases and severe electrolyte disturbances. In addition, 30 patients who came to our hospital for physical examination during the same period were selected as controls. The general conditions of the above three groups, such as age, gender, and body mass index, were not significant (all *P* < 0.05; Table 1).

**Table 1:** Comparison of general data in three groups

Features	Control group (*n* = 30)	ACI group (*n* = 52)	CCS group (*n* = 35)	*P*
Age (years)	64.47 ± 8.28	65.75 ± 7.16	65.20 ± 7.40	0.758
Gender (male)	17 (56.67%)	33 (63.46%)	24 (68.57%)	0.611
BMI (kg/m^2^)	23.18 ± 2.45	22.82 ± 2.87	23.67 ± 2.65	0.357
Hypertension (yes)	16 (53.33%)	27 (51.92%)	19 (54.29%)	0.976
Hyperlipoidemia (yes)	12 (40.00%)	25 (48.08%)	17 (48.57%)	0.735
Diabetes (yes)	7 (23.33%)	14 (26.92%)	10 (28.57%)	0.889
Heart rate (times/min)	83.37 ± 4.38	84.15 ± 3.03	85.23 ± 2.74	0.083
Systolic pressure (mmHg)	134.10 ± 18.16	140.67 ± 21.57	143.77 ± 18.09	0.139
Fasting blood glucose (mmol/L)	5.09 ± 1.67	6.39 ± 1.97*	6.65 ± 2.09*	0.003
Creatinine (μmol/L)	73.43 ± 5.60	127.49 ± 17.99*	131.08 ± 15.93*	<0.001
GCS score	−	6.33 ± 1.68	4.40 ± 1.72^#^	<0.001
APACHE Ⅱ score	−	10.88 ± 1.52	12.14 ± 1.33^#^	0.001
CK-MB (U/L)	10.74 ± 1.36	21.79 ± 3.14*	25.91 ± 3.86*^,#^	<0.001
NT-proBNP (μg/L)	117.21 ± 11.71	973.96 ± 277.34*	1330.47 ± 361.53*^,#^	<0.001
LVEF (%)	61.64 ± 2.26	52.57 ± 2.15*	53.34 ± 2.79*	<0.001
Etiological subtypes				0.060
LAA	−	19 (36.54%)	12 (34.29%)	
SAO	−	28 (53.85%)	13 (37.14%)	
CE	−	5 (9.61%)	10 (28.57)	

Note: ACI, acute cerebral infarction; CCS, cerebral-cardiac syndrome; BMI, body mass index; GCS: Glasgow coma scale; APACHE, Acute Physiology and Chronic Health Evaluation; CK-MB, creatine kinase-MB isoform; NT-proBNP, N-terminal probrain natriuretic peptide; LVEF, left ventricular ejection fraction; LAA, large-artery atherosclerosis; SAO, small-artery occlusion; CE, cardioembolism. Measurement data were compared by one-way analysis of variance and SNK-q test, and enumeration data were analyzed by chi-square test. *P < 0.05 vs the control group; #*P* < 0.05 vs the ACI group.

#### 2.1.1 General testing and determination of serum levels of HIF-1α and VEGF

The cubital venous blood (5 mL) was taken from all ACI patients on an empty stomach in the early morning at 1st, 3rd, and 7th days after admission and healthy volunteers from the control group, anticoagulated with sodium citrate, centrifuged, and stored at −80℃ for reserve. The fasting blood glucose (FBG), creatinine, and creatine kinase-MB isoform (CK-MB) levels of patients were measured by an OLYMPUS AU640 automatic biochemistry analyzer (Tokyo, Japan). A PHILIPS HD11 XE color Doppler ultrasound (Bothel, WA, USA) was utilized to evaluate the left ventricular ejection fraction (LVEF) of patients. Serum levels of N-terminal probrain natriuretic peptide (NT-proBNP), HIF-1α, and VEGF were measured by enzyme-linked immunosorbent assay (ELISA). Human NT-proBNP (ml061452), HIF-1α (ml058286), and VEGF (ml064281) ELISA kits were available from Shanghai Enzyme-Linked Biotechnology Co., Ltd. (Shanghai, China), and the specific steps were operated following the kit instructions.

### 2.2 Evaluation indicators

The Glasgow coma scale (GCS) score and Acute Physiology and Chronic Health Evaluation (APACHE) II score were implemented to assess the mental status and disease severity of patients after discharge [18,19]. On the 30th day after the onset of ACI, the National Institutes of Health Stroke Scale (NIHSS) and the modified Rankin Scale (mRS) score were utilized to evaluate the prognosis of patients [20]: an NIHSS score of ≤8 was considered to have a good short-term prognosis; NIHSS score >8 was considered a poor short-term prognosis; the mRS score ≤2 was defined as good short-term prognosis, and mRS score >2 was defined as poor short-term prognosis.

### 2.3 Statistical analysis

SPSS 21.0 statistical software was used for data analysis. Normally distributed measurement data were expressed as mean ± standard deviation, which were analyzed by one-way analysis of variance and SNK-q test. If normality was not satisfied, the data were transformed and then analyzed, and the HIF-1α and VEGF levels at different time points were compared by repeated measures analysis of variance. Enumeration data were compared using the chi-square test. The role of serum HIF-1α and VEGF levels in the prognosis judgment of CCS patients was assessed by receiver operating characteristic curve (ROC) analysis. The binary logistic regression analysis was implemented to analyze the independent risk factors influencing the prognosis of ACI combined with CCS. *P* < 0.05 was considered statistically significant.


**Ethical approval:** The research related to human use has been complied with all the relevant national regulations, institutional policies, and in accordance the tenets of the Helsinki Declaration and has been approved by the authors’ institutional review board or equivalent committee. This study complied with Declaration of Helsinki and was reviewed and approved by the ethics committee of First Affiliated Hospital of Hebei North University (Approval number: W2022027).
**Informed consent:** Informed consent has been obtained from all individuals included in this study.

## 3 Results

### 3.1 General data comparisons

The levels of FBG and creatinine in the ACI group were significantly higher than those in the control group, and the levels of which in the CCS group were significantly higher than those in the ACI and the control groups (all *P* < 0.05). The lower GCS score and higher APACHE II score were observed in the CCS group versus the ACI group (both *P* < 0.05). The elevated NT-proBNP and CK-MB and reduced LVEF were witnessed in the CCS and the ACI groups in compassion to the control group (all *P* < 0.05). Higher NT-proBNP and CK-MB were found in the CCS group in contrast to the ACI group (both *P* < 0.05) (Table 1).

#### 3.1.1 CCS patients have increased HIF-1α and VEGF levels

The serum HIF-1α level of patients in the ACI and the CCS groups showed a high rise at the 1st day, the 3rd day, and the 7th day after admission, and serum HIF-1α levels gradually showed a decreasing trend over time (all *P* < 0.05). The serum VEGF level of patients in the ACI and the CCS groups increased at the 1st day, the 3rd day, and the 7th day after admission and reached its peak at the 3rd day, but it was still higher than the control group (all *P* < 0.05) at the 1st day and the 7th day. In addition, the levels of serum HIF-1α and VEGF in the CCS group were significantly higher than the ACI group (both *P* < 0.05) (Table 2).

**Table 2:** Comparison of serum HIF-1 and VEGF levels in three groups

Group	HIF-1α (ng/mL)	VEGF (ng/mL)
Control group (*n* = 30)	125.63 ± 49.75	301.56 ± 110.47
**ACI group (*n* = 52)**		
1 day	391.07 ± 148.61*	697.32 ± 245.25*
3 days	329.58 ± 121.90*	753.49 ± 217.83*
7 days	276.54 ± 129.37*	658.76 ± 190.18*
**CCS group (*n* = 35)**		
1 day	537.68 ± 186.44*^,#^	991.54 ± 335.21*^,#^
3 days	482.49 ± 137.54*^,#^	1196.28 ± 261.47*^,#^
7 days	394.12 ± 151.58*^,#^	810.92 ± 214.08*^,#^

Note: ACI, acute cerebral infarction; CCS, cerebral-cardiac syndrome; HIF-1α, hypoxia-inducible factor-1α; VEGF, vascular endothelial growth factor. HIF-1 and VEGF levels at different time points were compared using repeated measures analysis of variance. *P < 0.05 vs the control group; ^#^ *P* < 0. 05 vs the ACI group at the same time point.

#### 3.1.2 The relationship between clinical data, HIF-1α, and VEGF levels and the prognosis of patients with CCS

The NIHSS or mRS score evaluated that on the 30th day after discharge, patients were divided into a good prognosis group and a poor prognosis group. Based on the NIHSS score of ≤8 or >8, we divided 35 CCS patients into 19 cases (54.29%) in the good prognosis group and 16 cases (45.71%) in the poor prognosis group. Next, the difference parameters in the general data of the two groups, including FBG, creatinine, GCS score, APACHE II score, CK-MB, NT-proBNP, LVEF, and HIF-1α, and VEGF levels at the 7th day of admission, were analyzed. The findings demonstrated that FBG, APACHE II score, CK-MB, and HIF-1α and VEGF levels at the 7th day of admission were higher while GCS score was lower in the poor prognosis group than those in the good prognosis group (all *P* < 0.05; Table 3).

**Table 3:** The relationship among clinical data, HIF-1α and VEGF levels and prognosis of CCS patients (according to NIHSS score)

	Good (*n* = 19)	Poor (*n* = 16)	*t*/*χ* ^2^	*P*
Fasting blood glucose	5.94 ± 1.86	7.50 ± 2.07	2.352	0.025
Creatinine	126.88 ± 17.22	136.06 ± 13.06	1.748	0.090
GCS	5.00 ± 2.00	3.69 ± 0.95	2.404	0.022
APACHE Ⅱ	11.68 ± 1.06	12.69 ± 1.45	2.367	0.024
CK-MB	24.41 ± 3.31	27.69 ± 3.79	2.729	0.010
NT-proBNP	1242.67 ± 347.77	1434.73 ± 360.23	1.601	0.119
LVEF	53.80 ± 2.96	52.79 ± 2.56	1.073	0.291
HIF-1α (7 days)	302.95 ± 114.20	502.39 ± 115.75	5.115	<0.001
VEGF (7 days)	694.49 ± 174.05	949.18 ± 173.11	4.323	<0.001
Etiological subtypes			3.393	0.183
LAA (*n* = 12)	8 (42.10%)	4 (25.00%)		
SAO (*n* = 13)	8 (42.10%)	5 (31.25%)		
CE (*n* = 10)	3 (15.80%)	7 (43.75%)		

Note: CCS, cerebral-cardiac syndrome; NIHSS, National institutes of health stroke scale; HIF-1α, hypoxia-inducible factor-1α; VEGF, vascular endothelial growth factor; GCS: Glasgow coma scale; APACHE, Acute Physiology and Chronic Health Evaluation; CK-MB, creatine kinase-MB isoform; NT-proBNP, N-terminal probrain natriuretic peptide; LVEF, left ventricular ejection fraction; LAA, large-artery atherosclerosis; SAO, small-artery occlusion; CE, cardioembolism.

Similarly, based on the mRS score of ≤2 or >2, we divided 35 patients with CCS patients into 21 cases (60.00%) in the good prognosis group and 14 patients (40.00%) in the poor prognosis group. The findings disclosed that FBG, CK-MB, and HIF-1α and VEGF levels at the 7th day of admission were higher while GCS score was lower in the poor prognosis group than those in the good prognosis group (all *P* < 0.05; Table 4).

**Table 4:** The relationship among clinical data, HIF-1α and VEGF levels and prognosis of CCS patients (according to mRS score)

	Good (*n* = 21)	Poor (*n* = 14)	*t*/*χ* ^2^	*P*
Fasting blood glucose	5.93 ± 1.80	7.74 ± 2.08	2.738	0.010
Creatinine	127.18 ± 16.37	136.91 ± 13.80	1.831	0.076
GCS	4.95 ± 1.96	3.57 ± 0.76	2.503	0.017
APACHE Ⅱ	11.81 ± 1.12	12.64 ± 1.50	1.880	0.069
CK-MB	24.75 ± 3.32	27.65 ± 4.07	2.319	0.027
NT-proBNP	1246.44 ± 330.75	1456.52 ± 380.82	1.733	0.092
LVEF	53.96 ± 2.91	52.41 ± 2.39	1.657	0.107
HIF-1α (7 days)	320.73 ± 122.70	504.20 ± 123.21	4.326	0.001
VEGF (7 days)	726.33 ± 191.95	937.81 ± 185.14	5.086	<0.001
Etiological subtypes			5.337	0.069
LAA (*n* = 12)	9 (42.86%)	3 (21.43%)		
SAO (*n* = 13)	9 (42.86%)	4 (28.57%)		
CE (*n* = 10)	3 (14.28%)	7 (50.00%)		

Note: CCS, cerebral-cardiac syndrome; mRS, modified Rankin Scale; HIF-1α, hypoxia-inducible factor-1α; VEGF, vascular endothelial growth factor; GCS: Glasgow coma scale; APACHE, Acute Physiology and Chronic Health Evaluation; CK-MB, creatine kinase-MB isoform; NT-proBNP, N-terminal probrain natriuretic peptide; LVEF, left ventricular ejection fraction; LAA, large-artery atherosclerosis; SAO, small-artery occlusion; CE, cardioembolism.

### 3.2 HIF-1α and VEGF levels in the prognosis evaluation of CCS

**Figure 1:**
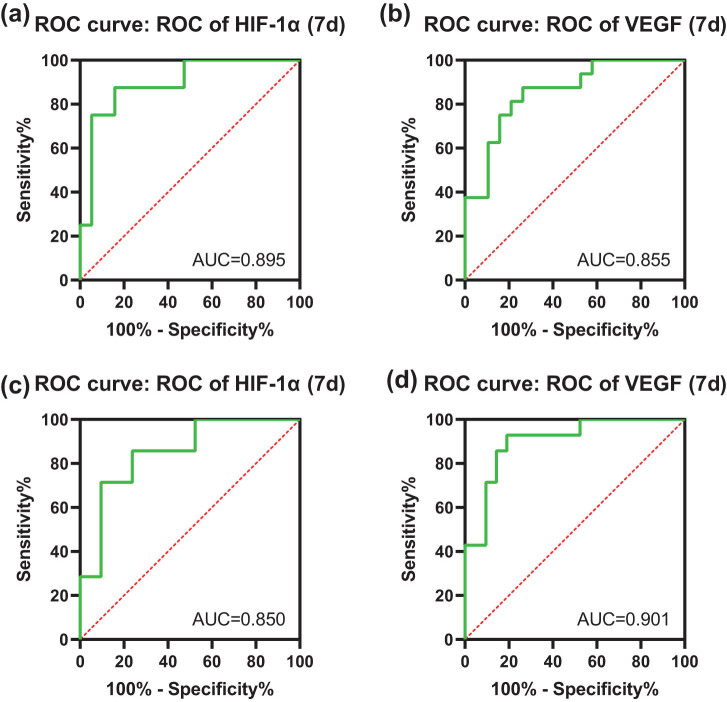
The roles of serum HIF-1 and VEGF levels in determining CCS prognosis by ROC. According to the prognosis of the NIHSS score, the ROC was utilized to evaluate the roles of the serum HIF-1α (a) and VEGF (b) levels. According to the prognosis of the mRS score, the ROC was utilized to evaluate the roles of the serum HIF-1α (c) and VEGF (d) levels.

Next, the ROC was drawn to evaluate the roles of serum HIF-1 and VEGF levels in determining CCS prognosis (Figure 1 and Table 5). According to the prognosis of the NIHSS score (Figure 1a and b and Table 5), the area under the curve (AUC) of serum HIF-1α level for predicting poor prognosis in patients with ACI combined with CCS was 0.895, with an optimal cut-off value of 391.58 ng/mL, a sensitivity of 87.5%, and a specificity of 84.2%. The AUC of serum VEGF level for predicting poor prognosis in patients with ACI combined with CCS was 0.855, with an optimal cut-off value of 749.025 pg/mL, a sensitivity of 87.5%, and a specificity of 73.7%. According to the prognosis of the mRS score (Figure 1c and d and Table 5), the AUC of serum HIF-1α level for predicting poor prognosis in patients with ACI combined with CCS was 0.850, with an optimal cut-off value of 391.58 ng/mL, a sensitivity of 85.7%, and a specificity of 76.2%. The AUC of serum VEGF level for predicting poor prognosis in patients with ACI combined with CCS was 0.901, with a cut-off value of 798.21 pg/mL, a sensitivity of 92.9%, and a specificity of 81.0%. The AUC was greater than 0.8 in the results of ROC curve analysis in all four groups, indicating that serum levels of HIF-1α and VEGF may be valuable markers in predicting prognosis in patients with ACI combined with CCS.

**Table 5:** Results of ROC curve analysis

	Indicator	Sensitivity (%)	Specificity (%)	Positive predictive value (%)	Negative predictive value (%)	Area under the curve	Optimal cut-off value
A	HIF-1α	87.5	84.2	82.4	88.9	0.895	391.58 ng/mL
B	VEGF	87.5	73.7	73.7	87.5	0.855	749.025 pg/mL
C	HIF-1α	85.7	76.2	70.6	88.9	0.850	391.58 ng/mL
D	VEGF	92.9	81.0	76.5	94.4	0.901	798.21 pg/mL

Note: HIF-1α, hypoxia-inducible factor-1α; VEGF, vascular endothelial growth factor.

Finally, we included possible factors affecting the prognosis of patients with ACI combined with CCS, such as FBS, creatinine, GCS score, APACHE II score, CK-MB, NT-proBNP, LVEF, etiological subtypes, and HIF-1α and VEGF levels at the 7th day of admission in a binary logistic regression analysis (forwarded: LR), which revealed that HIF-1α and VEGF levels (both OR > 1, *P* < 0.05, Table 6) may be independent risk factors influencing the prognosis of ACI combined with CCS.

**Table 6:** Multi-factor logistic regression analysis of prognosis in patients with ACI combined with CCS

	*β*	S.E.	Wals	*P*	OR	95% CI
**Prognosis according to NIHSS scores**						
HIF-1α	0.022	0.009	5.991	0.014	1.022	1.004–1.04
VEGF	0.009	0.004	4.798	0.028	1.009	1.001–1.017
Constant (quantity)	−16.406	5.865	7.825	0.005	0	
**Prognosis according to mRS scores**						
HIF-1α	0.017	0.008	4.691	0.030	1.017	1.002–1.033
VEGF	0.012	0.005	6.348	0.012	1.012	1.003–1.022
Constant (quantity)	−18.252	6.552	7.761	0.005	0	

Note: ACI, acute cerebral infarction; CCS, cerebral-cardiac syndrome; NIHSS, National institutes of health stroke scale; mRS, modified Rankin Scale; HIF-1α, hypoxia-inducible factor-1α; VEGF, vascular endothelial growth factor; *β*, regression coefficient; S.E., standard error; OR, odds ratio; CI, confidence interval.

## 4 Discussion

It has been reported that CCS can be found in 25–75% ACI patients, depending on diverse study design and examine regimens [7]. CCS has a great effect on the morbidity, mortality, and prognosis of ACI and ranks as the second leading reason of death after cerebrovascular disease [8]. CCS is now receiving more attention due to the novel effects of stroke on cardiac physiophysiological comorbidity. Here, in this research, we aimed to discuss the recent prognosis of patients with ACI combined with CCS by examining the roles of HIF-1 and VEGF levels.

VEGF is currently considered a powerful and highly specific angiogenic factor [21]. Specifically, VEGF is mainly implicated in the neurovascular remodeling in the ischemic brain, and it enhances angiogenesis and brain plasticity, protects the neurons from injury, and improves the neural precursor cell recruitment and proliferation [22]. Evidence has demonstrated that VEGF expression is elevated in the ischemic penumbra and the surrounding region after ACI [23]. In our study, higher serum VEGF level was observed in the CCS and ACI groups versus the control group, and its level was even higher in the CCS group in comparison to the ACI group. In the process of cerebral ischemia, HIF-1α‑induced gene expression is able to induce reperfusion of the ischemic penumbra area, thereby improving glucose transport and strengthening the ability of the cells to tolerate hypoxia, which is protective for ischemic and hypoxic neurons [24,25]. A recent study has pointed out that elevated serum HIF-lα levels after aneurysmal subarachnoid hemorrhage (aSAH) are in independent association with stroke severity and are independently linked with delayed cerebral ischemia and 6-month poor outcome, implying that serum HIF-lα could serve as a potential prognostic biomarker of aSAH [26]. In our study, higher serum HIF‐1α level was observed in the CCS and ACI groups versus the control group, and its level was even higher in the CCS group in comparison to the ACI group. In line with our findings, there are some articles highlighting the same trends of HIF‐1α and VEGF in brain-associated diseases. For instance, Cramer et al. have reported that HIF-induced increase of VEGF expression could be a mechanism for the post-ischemic neurogenesis in the brain [27]. Sun et al. have stated that high HIF-1α level modulates cytokines expressing VEGF in hypoxia ischemia region and enhances the formation of new blood vessels, thereby serving a protective role in the brain tissue in early ACI [28]. Nevertheless, the serum levels of HIF-1 and VEGF in ACI combined with CCS need further validation.

NIHSS is one of the most important scales for evaluating the neurological function in ACI patients, and it could effectively reflect the neurological deficits of patients and accurately determine their prognosis [29,30]. The mRS is commonly applied to assess the disability of ACI patients or other reason of neurological disability and it is also utilized as an end point for the clinical trials [31]. A recent article has disclosed that NIHSS and mRS scores have a close relation with the prognosis of ACI. The higher NIHSS and mRS scores reflect the worse prognosis of patients [3]. Based on this, in our research, the NIHSS and the mRS score were utilized to evaluate the prognosis of patients with ACI combined with CCS. The corresponding findings demonstrated that according to the prognosis of the NIHSS score, FBG, APACHE II score, CK-MB, and HIF-1α and VEGF levels at the 7th day of admission were higher while GCS score was lower in the poor prognosis group than those in the good prognosis group, and the AUC of serum HIF-1α and VEGF levels was 0.895 (95% confident interval [CI], 0.786–1.000) and 0.855 (95% CI, 0.731–0.980). According to the prognosis of the mRS score, FBG, CK-MB, and HIF-1α and VEGF levels at the 7th day of admission were higher while GCS score was lower in the poor prognosis group than those in the good prognosis group, and the AUC of serum HIF-1α and VEGF levels was 0.850 (95% CI, 0.722–0.979) and 0.901 (95% CI, 0.798–1.000). All these results confirmed that HIF-1α and VEGF levels in serum may be a meaningful prognostic symbol of CCS patients. Furthermore, the results of the binary logistic regression analysis revealed that HIF-1α and VEGF levels may be independent risk factors influencing the prognosis of ACI combined with CCS.

In summary, we observed that the serum levels of HIF-1α and VEGF were upregulated in CCS patients compared with healthy controls and ACI patients. In addition, the levels of HIF-1α and VEGF in the serum of CCS patients with poor prognosis were higher than those of CCS patients with good prognosis. HIF-1α and VEGF levels may be meaningful prognostic markers in CCS patients. However, this study was a retrospective analysis with a small sample size, and a prospective multicenter clinical study with a large sample size is still needed to make the results more representative. In addition, this study investigated the roles of HIF-1α and VEGF levels in the short-term prognosis of patients with ACI combined with CCS, and whether there is a guiding effect on the long-term prognosis is to be found subsequently. Future research will be conducted to study the relationship and relevance of the impact of small vessel disease versus other ischemic subtypes at the level of sigma due to the fact that the pathophysiology, prognosis, and clinic of ischemic small vessel strokes are different from the rest of cerebral infarcts [32]. It is also expected that an animal model of ACI combined with CCS will be established in the future, and its therapeutic effect on ACI combined with CCS will be investigated by treatment with HIF-1α inhibitor (2ME2) or anti-VEGF neutralizing antibody (RB-222) injected into the lateral ventricle.
